# Hepatitis B infection in the rural area of Dschang, Cameroon: seroprevalence and associated factors

**DOI:** 10.11604/pamj.2020.36.362.17787

**Published:** 2020-08-28

**Authors:** Thomas Djifack Tadongfack, François Roger Nguepy Keubo, Patrice Bianke

**Affiliations:** 1 Saint Vincent de Paul Hospital Dschang, Dschang, Cameroon,; 2 University of Dschang, Dschang, Cameroon

**Keywords:** Viral Hepatitis B, seroprevalence, associated factors, Dschang, Cameroon

## Abstract

**Introduction:**

viral hepatitis B remains a major public health problem around the world, especially in underdeveloped and developing countries. Cameroon shows a grate variability in prevalence of this infection in the country and even within different populations groups. The aim of this study was to determine the prevalence and factors associated with viral hepatitis B infection in the rural area of Dschang.

**Methods:**

we conducted a cross-sectional community-based study, involving 551 participants of both genders recruited by a voluntary sampling technique. The biological diagnosis of HBsAg was done by the Immunochromatographic method (PKL® kit of PARAMEDICAL srl laboratories). Positive cases were confirmed by ELISA method (reagent Kit from DIALAB Laboratories).

**Results:**

results show a Viral Hepatitis B prevalence of 5.08% (95% CI: 3.2-6.9). University students were the most infected (11/88) with a positivity rate of 12.50% (95%CI: 5.6-19.4). Only 29/551 participants (5.26%) had received at least one dose of vaccine against the disease and were less infected (3.44%) than the others (5.17%). Age (p=0.000), level of education (p=0.013), occupation (p = 0.002), belief in the traditional healing of hepatitis B (p=0.000) and knowledge about the disease and its contamination roads (p=0.049) were associated with viral hepatitis B.

**Conclusion:**

there is a need of intensifying awareness, education, routine screening and vaccination of the population, especially in schools and university milieu to better counteract the infection with hepatitis B virus in our local Communities.

## Introduction

Hepatitis B infection remains a serious public health problem in several parts of the globe. It is the major cause of liver cirrhosis and hepatocellular carcinoma [1,2]. There is a continuous increase death rate due to this pathology. WHO estimated in 2015 at approximately 257 million people suffering from a chronic form of Hepatitis B virus (HBV) infection, with close to 1.34 million deaths. This figure is comparable to deaths related to tuberculosis and greater than those caused by HIV / AIDS [2]. While sub-Saharan Africa and Western Pacific regions called high endemic regions are mostly affected by this epidemic, others like the United State, the Northern Europe, Australia and parts of Latin America present prevalence rates below 2.00% [1,3]. Some areas also witness moderate rates with prevalence oscillating between 2.00% and 8.00 [4-6]. Until 2004 in France, immunization coverage against hepatitis B among school children and adolescents (33.00% - 42.00%) and more problematic [7].

In sub-Saharan Africa, the prevalence rate of HBV infection is between 8.00 and 20.00% [8]. This high prevalence is probably due to its transmission routes which are mainly vertical, from mother to newborn at birth, and horizontal through contacts with infected persons, especially in the perinatal period [9]. Cameroon witness a wide disparity in the prevalence of HBV infection within the country. Noah et al, in a study carried out in general population in 2015 reported variable rates per region; ranging from 22.82% in the Far North, 21.53% in the North, 12.75% in the Adamawa, 14 .00% in the East and 5.22% in the South for an overall prevalence of 13.01% [10]. Numerous other studies reported prevalence rates among health care professionals of 4.98% in the Fako division of the south west region [11] and 11.00% in Yaoundé [12]. Since the implementation of the mother to child transmission prevention strategy against hepatitis B (PMTCT-HBV) in Cameroon [13], studies have been carried out in order to better control the outcome most often fatal in children [13,14]. These initiatives are of valuable importance in view of the high probability of chronic carriage in children [15]. Dionne-Odom et al. in a study conducted among blood donors in four large Hospitals of the Cameroon Baptist Convention Health Services reported a 4.40% prevalence of HBV among blood donors [14]. Thus, underlining the relevance of the screening of Hepatitis B surface Antigen (HBsAg) in pre-transfusion situation. In view of the multiplicity of factors contributing to the resurgence of HBV infection in developing countries, continuous and efficient measures need to be implemented. WHO in the Global hepatitis report, 2017 target the elimination of this infection as a threat to public health by 2030 [1]. This study aims at determining prevalence of hepatitis B infection and associated factors in the rural area of Dschang in order to intensify awareness, preventive measures and the need of proper immunization.

## Methods

**Material**: we conducted a cross-sectional community-based study, with Saint Vincent de Paul Hospital Dschang (SVPHD) as recruitment site. A total of 551 participants of both genders were enrolled in the study through a screening campaign against HBV and vaccination, organized by the hospital between May and June 2018. Were included in the study participants voluntarily arriving the recruitment site following sensitization through one of the communication communication channels used (posters, churches, community radio, community associations and relatives). Following registration of each participant, a structured questionnaire was used to collect the data after reading the information leaflet and signing the free informed consent form. A volume of approximately 4-5 mL of blood was aseptically collected on an EDTAK3 (Ethylene Diamine Tetra-acetic Tri-Potassium) tube in a specially designed space and sent to the hospital laboratory for analysis after centrifugation.

**Method**: plasma was used for the detection HBsAg by immunochromatographic method with the PKL^®^kit of PARAMEDICAL srl laboratories (Corso Vittorio Emanuele 127-84123 Salerno (SA) Italy). The analysis was performed according to the manufacturer's instructions. Positive results on immunochromatography were further confirmed by the ELISA method using the DIALAB^®^Laboratories kit (DIALAB, A-2351 Neudorf Austria). Positivity was established for sample with an absorbance greater than 1. Each participant tested negative was referred to the SVPHD vaccination unit for registration and hepatitis B vaccine administration, while those tested positives were referred by clinician to the hepatologist for further assessment and follow up. The data collected were compile using Microsoft Excel 2016 spreadsheets and then transferred into SPSS software version 25.0 (SPSS Inc., Chicago, USA) for cleaning and analysis. Prevalence specific to each characteristic were calculated and expressed with their 95% confidence interval (CI), medians with their percentiles (25-75). The Chi^2^ and Fisher exact tests were used to compare the categorical variables and to evaluate the association with the factors considered for a significance level of 5%.

## Results

Table 1 summarizes some characteristics of the study population. Female participants were more represented than male with respective proportions of 62.61% and 37.39%. The median age of participants was 25 (14-44 years). The Prevalence of viral hepatitis B (HBsAg +) was 5.08% (95%CI: 3.2-6.9), with 4.35% (95%CI: 2.2-6.5) female participants and 6.31% (95%CI: 3.0-9.6) male participants infected in the respective groups. The age group [17-19] was the most affected by the HBV infection with a positivity rate of 12.64% (95%CI: 7.7-17.6). Only 29/551 participants (5.26%) had received at least one dose of vaccine against the disease and were less infected (3.44%) than others (5.17%). There was no significant difference between the immunization status and HBV infection (p > 0.05). According to the occupation of participants, the disease was predominant among university students, with a prevalence of 12.50% (95% CI: 5.6-19.4). Despite the fact that participants with University level of education had the highest knowledge about the infection (48.58%, p=0.000) among the different levels of education (Table 2), they were more infected with a positivity rate of 10.00% (95 % CI: 5.4-14.6). Of the study population, respondents who admitted a possibility of treatment of this pathology by the traditional healers presented a positivity rate of 15.49% (95% CI: 7.1-23.9), more than 4 times greater than 3.54% (95% CI: 1.9-5.2) obtained in the other group. Age (p = 0.000), level of education (p = 0.013), occupation (p = 0.002), traditional treatment of hepatitis B (p=0.000) and knowledge about the disease and its modes of contamination (p = 0.049) were associated with HBV infection. There was no statistically significant difference (p> 0.05) with respect to the other variables evaluated. Although there was no association between the community surroundings and the distribution of infection, participants having affected people in their surroundings in the community show a positivity rate of 8.00%, almost 2 times higher than that (4.79%) obtained among participants living with a surrounding of uninfected persons (Table 3).

**Table 1:** sociodemographic characteristics of the study population

Characteristics	Number	Frequency
**Age groups (years)**		
2 – 16	179	**32.49 %**
17 – 31	174	31.58 %
32 – 46	87	15.79 %
47 – 60	81	14.70 %
> 60	30	5.44 %
**Gender**		
Male	206	37.39%
Female	345	62.61%
**Level of education**		
Illiterate	19	3.45 %
Primary	151	2 7. 4 0 %
Secondary	221	**40.11 %**
Superior	160	29.04 %
**Occupation**		
University student	88	15.97 %
Below university	219	**39.75 %**
Housewife	62	11.25 %
Trader	27	4.9 0%
Farmer	26	4.70 %
Teacher	77	13.97 %
Others	52	9.4 4%
**Marital status**		
Single	328	**59.53 %**
Married	198	35.93 %
Divorced	2	0.36 %
Widow	23	4.17 %
**Total**	**551**	

**Tableau 2:** knowledge on the disease and contamination routes according to the level of education

Level of education	Knowledge on the disease and contamination route		P-value
	Yes **N=247** (44.83%)	No **N=304** (55.17%)	
Illiterate	5 (2.02%)	14 (4.61%)	0.000^***^
Primary	30 (12.15%)	121 (21.96%)	
Secondary	92 (37.25%)	129 (23.41%)	
University	120 (48.58%)	40 (7.26%	

**Table 3:** distribution of viral hepatitis B (HBsAg +) according to the factors evaluated

Characteristics	N-positive / N-tested	Prevalence	95% CI	p-value
**Age groups (years)**				
2 – 16	1/179	1.45%		0.000 ^***^
17 – 31	22/174	**12.64%**	**7.7 - 17.6**	
32 – 46	3/87	3.44%		
47 – 60	2/81	2.46%		
> 60	0/30	0.00%		
**Level of education**				
Illitrate	1/19	5.26%		0.013 ^*^
Primary	4/151	2.64%		
Secondary	7/221	3.16%		
Superior	16/160	**10.00%**	**5.4 - 14.6**	
**Occupation**				
University students	11/88	**12.50%**	**5.6 - 19.4**	0.002 **
Bellow university	3/219	1.36%		
Housewife	5/62	8.06%		
Trader	2/27	7.40%		
Farmer	0/26	0.00%		
Teacher	5/77	6.49%		
Others	2/52	3.84%		
**Marital status**				
Single	20/328	**6.09%**	**3.5 - 8.7**	0.465
Married	8/198	4.04%		
Divorced	0/2	0.00%		
Widow	0/23	0.00%		
**Surroundings of Hepatitis B positive persons**				0.308
Yes	4/50	**8.00%**	**0.5 - 15.5**	
No	24/501	4.79%		
**Vaccinated against Hepatitis B**				1.000
Yes	1/29	3.44%		
No	27/522	**5.17%**	**3.3 - 7.1**	
**Belief in traditional healing of Hepatitis B**				0.000 ^***^
Yes	11/71	**15.49%**	**7.1 - 23.9**	
No	17/480	3.54%		
**Knowledge on the disease and contamination routes**				0.049 ^*^
Yes	18/247	**7.28%**	**4.0 - 10.5**	
No	10/304	3.28%		
**General prevalence**	**28/551**	**5.08%**	**3.2 - 6.9**	

N = number, ^***^: p <0.001 , ^**^: p <0.01, ^*^: p <0.05

## Discussion

Viral Hepatitis B remains a pathology in sharp increase in the Cameroon society, and particularly in a context where access to adequate health care remains a privilege of a minority. Numerous public health programs with both short, medium and long term objectives are being implemented in communities on daily bases [1,16]. Particularly in highly endemic areas with the aim of better controlling the distribution of HBV infection. The present study aimed at determining the prevalence of this infection and some possible associated factors in the rural area of Dschang in Cameroon to intensify awareness, preventive measures, and the need of proper immunization in the global context. The distribution of HBV was found to be 5.08 % in the present study carried out in the population of Dschang. This result is higher than the 4.1% recorded by Mbopi-keou *et al*. [17] in a study conducted in Bafoussam, Cameroon. This disparity can be justified by the difference in the population studied, of which those in the present one were mostly students who are more likely to be vulnerable to unsafe sexual practices. Also, Noah *et al*. [10] reported a higher prevalence of 22.82% within the general population in the extreme north of Cameroon. This difference may be due, on the one hand, to the level of knowledge of study participants, which is higher than that of Cameroonians in the northern part of the country, and also to the questions of sex education that remains taboo in that part of the country.

The age group [17-19] years was the most affected by HBV with a positivity rate of 12.64%. This result disagrees with that of Ageely *et al*. [4] who found in a study conducted in the Jazan region of Saudi Arabia that the most affected age group was those over 60 years old, with an occurrence of 22.20 %. This disparity can be justified by the difference in contexts in which both studies were conducted. Indeed, there would be a difference in age groups where individuals are more sexually active in both societies. If in our context young people are more sexually active, in Arab society we would probably attend the opposite. In other words, the sexual activity would be reserved for people with certain maturity. Efforts in the fight against Hepatitis B increasingly value the primary prevention, with emphasis on vaccination of non-carriers of the virus. Only 29/551 participants (5.26%) reported having already received at least one dose of vaccine against the disease and were less infected (3.44%) than those not vaccinated at all (5.17%). Although not negligible, it can be noted that this proportion remains relatively low with reference to the goal set by WHO [1]. That notwithstanding, since immunization contributes to the reduction of the infection rate, as the results of this study tend to demonstrate, it would be judicious to encourage our communities in the way towards vaccination as recommended by WHO. Tatsilong *et al*. [12] in Yaoundé and Antona *et al*. [7] in France reported respective vaccination coverage of 19.00 % for health personnel and 33.00%- 43.00% for children and adolescents in school settings. Referring to these results, it can be emphasized that awareness-raising on the means of prevention and especially vaccination in the community should be quite consistent and focused.

A cross reading of the results shows that the highest prevalence of Hepatitis among occupation groups was 12.50% and attributed to university students. This rate is much higher than the 7.6 0 % obtained by Bagnaka *et al*. [18] among Medical and Pharmacy students of the University of Douala. Although there is little similarity in these two study populations (strictly student and community), these rates remain high. From the various databases assessed in this work, we did not find studies that categorized the distribution of HBV infection according to the level of education in Cameroon. However, the highest prevalence rate among participants with university-level of education is questionable in view of their greater ability to learn about Hepatitis B in this group of participants. This high rate could be justified by promiscuity and a multiplicity of sexual partners among university students who constitute the majority (84/160) of participants with university level of education in Dschang. Factors associated with HBV can vary from one context to another. Efficient infection control protocols are inspired from these factors. Several associated factors were identified in the present study. For instance, age (p=0.000) and level of education (p=0.013). Indeed, from the pubertal period, the prevalence decreases as the age increases. These results are contrary to those of Dovonou *et al*. [19] who did not find an association between these variables and HBV infection in their study conducted in HIV positive patients in Parakou, Benin. This dissimilarity may be due to the difference in age ranges and socio-cultural context between the studied populations.

The study also shows an association between occupation and HBV (p=0.002), unlike Nguekeng *et al*. [11] who showed that profession (Medical or Paramedical) was not associated with the prevalence of Hepatitis B surface antigen (HBsAg) among health care workers of the Fako Health District. This disparity may be justified by the diversity of occupations of the participants in the present study, which makes them vulnerable to the infection. As reported by Dovonou *et al*. [19], there was no statistically significant association between the disease and marital status. There was a reverse association between knowledge about the disease and its modes of contamination and morbidity to viral hepatitis B. The more participants were instructed about the infection, the more they are at risk of contracting the disease (p=0.049). This situation would be related to the fact that our sample population consisted mainly of university students who are for the majority in the sexually active age group.

## Conclusion

The prevalence of viral hepatitis B in Dschang (5.08%) remains below the National prevalence, but among the highest in the west region as compare to that obtained in Bafoussam. The highest infection rate is attributed to the youth group consisting mainly of university students. Belief in traditional healing of hepatitis B promotes the spread of infection in this rural area. This call for awareness, screening and vaccination of the population, mainly in school and university milieu for a better control of the infection with hepatitis B virus.

### What is known about this topic

- Viral hepatitis B is in upsurge worldwide and mainly in underdeveloped and developing regions;
- Its distribution is greatly variable in the 10 regions of Cameroon and among different population groups.

### What this study adds

- Prevalence of viral Hepatitis B in the rural area of Dschang and identification of the most affected group (here the university students);
- Highlighting a very low immunization coverage against viral hepatitis B in the Community of Dschang, Cameroon;
- Belief in the traditional healing of viral hepatitis B promotes its distribution in rural areas where the culture is still ingrained in the habits of the populations.
